# Phosphorylation of hnRNP A1–Serine 199 Is Not Required for T Cell Differentiation and Function

**DOI:** 10.4049/immunohorizons.2300074

**Published:** 2024-02-09

**Authors:** Tristan L. A. White, Ye Jin, Sean D. A. Roberts, Matthew J. Gable, Penelope A. Morel

**Affiliations:** Department of Immunology, University of Pittsburgh School of Medicine, Pittsburgh, PA

## Abstract

hnRNP A1 is an important RNA-binding protein that influences many stages of RNA processing, including transcription, alternative splicing, mRNA nuclear export, and RNA stability. However, the role of hnRNP A1 in immune cells, specifically CD4^+^ T cells, remains unclear. We previously showed that Akt phosphorylation of hnRNP A1 was dependent on TCR signal strength and was associated with Treg differentiation. To explore the impact of hnRNP A1 phosphorylation by Akt on CD4^+^ T cell differentiation, our laboratory generated a mutant mouse model, hnRNP A1-S199A (A1-MUT) in which the major Akt phosphorylation site on hnRNP A1 was mutated to alanine using CRISPR Cas9 technology. Immune profiling of A1-MUT mice revealed changes in the numbers of Tregs in the mesenteric lymph node. We found no significant differences in naive CD4^+^ T cell differentiation into Th1, Th2, Th17, or T regulatory cells (Tregs) in vitro. In vivo, Treg differentiation assays using OTII-A1-Mut CD4^+^ T cells exposed to OVA food revealed migration and homing defects in the A1-MUT but no change in Treg induction. A1-MUT mice were immunized with NP^−^ keyhole limpet hemocyanin, and normal germinal center development, normal numbers of NP-specific B cells, and no change in Tfh numbers were observed. In conclusion, Akt phosphorylation of hnRNP A1 S199 does not play a role in CD4^+^ T cell fate or function in the models tested. This hnRNP A1-S199A mouse model should be a valuable tool to study the role of Akt phosphorylation of hnRNP A1-S199 in different cell types or other mouse models of human disease.

## Introduction

Naive CD4 T cells differentiate into various Th cell subsets such as Th1, Th2, Th17, and T follicular helper (Tfh) cells and Tregs in response to either foreign or self-antigen (1–3). The differentiation of naive CD4^+^ T cells into specific Th subsets is determined by the nature of the pathogen and the signals received by the T cells. Thus, the ability to differentiate into appropriate Th subsets is important for immune responses in many disease models. For example, oral tolerance to food Ags and commensal bacteria is maintained by the differentiation of specific Tregs (4–10) in the intestine that regulate the innate and adaptive immune systems. Another example is Tfh induction in germinal center (GC) differentiation following immunization. Tfh cells are required for the formation of GC and the selection of GC B cells. The GC is a specialized microstructure that forms in the spleen in response to immunization, in which specific Tfh and B cells interact to produce high-affinity memory B cells and long-lived plasma cells, important for protection from reinfection (11, 12).

Understanding the mechanisms underlying T cell differentiation is essential for developing targeted therapies. The PI3K/Akt/mTOR signaling pathway is essential for the regulation of cell metabolism, growth, and survival (13, 14) and is tightly regulated by Phosphatase and Tensin Homolog (15), making it a potential target for immunotherapies (11). Activation of the PI3K/Akt/mTOR pathway in T cells is dose-dependent and has an impact on T cell fate (16–19). Stimulating naive T cells with a low dose of a high-affinity Ag fails to fully activate the PI3K/Akt/mTOR signaling pathway (17) and favors Treg fate (16, 20) and Th2 cell differentiation (21–23), whereas high Ag dose stimulation fully activates the PI3K/Akt/mTOR pathway, and this favors inflammatory Th1 cell differentiation (16, 17, 19, 22). The PI3K/Akt/mTOR signaling strength determines Akt phosphorylation state and substrate activity. When Akt is fully activated following high-dose stimulation, Akt is phosphorylated at two sites: Ser^473^ and Thr^308^ (24). In this state, Akt phosphorylates substrates such as Foxo1 (18), which has been shown to inhibit Treg induction (16, 18–25). Under low-dose Ag stimulation, Akt is only phosphorylated at Thr^308^, and the lack of phosphorylation at Ser^473^ results from a reduction in mTORC2 activity (26) We have shown that Akt phosphorylates different sets of substrates under these two activation conditions, and we observed the phosphorylation of hnRNP A1 and other RBPs in Treg-inducing conditions (18). Furthermore, siRNA knockdown of hnRNP A1 inhibited the generation of Treg induced under low-dose conditions (18). Previous studies have shown that Akt interacts with hnRNP A1 (27) and that Akt phosphorylates hnRNP A1 at Ser^199^ (27, 28). The mechanism by which Akt phosphorylation of hnRNP A1 affects T cell differentiation is unclear.

hnRNPs are RNA-binding proteins that play crucial roles in RNA biogenesis, expression, and function (29). Sequencing of the human genome and transcriptome has revealed that more than 90% of multiexon genes are alternatively spliced in a tissue-specific manner. In cell lines, Akt phosphorylation of hnRNP A1 has been shown to modulate hnRNP A1 function (27, 29). hnRNP A1 is one of the most abundant and well studied RNA-binding proteins known to play a role in transcription, splicing, and RNA stability (29). The function of hnRNP A1 is not limited to mRNA biogenesis. When hnRNP A1 is exported out of the nucleus, it undergoes several post-translational modifications, including phosphorylation, ubiquitination, and methylation, which regulate its activity. hnRNP A1 is known to shuttle between the nucleus and cytoplasm, affecting multiple stages of RNA biogenesis (29). The goal of this study was to determine the role of Akt phosphorylation of hnRNP A1 in naive CD4^+^ T cell differentiation, Tregs induction/function oral tolerance, and Tfh and B cells’ ability to form a GC reaction.

## Material and Methods

### Generation of Hnrnpa1-S199A mutant mice

The Hnrnpa1-S199A mutation was created using CRISPR/Cas9 by the Innovative Technologies Development Core at the University of Pittsburgh. To introduce the mutation in the locus, we used a 150-bp single-stranded oligodeoxyribonucleotide (ssODN) ultramer (Innovative Technologies Development Core) as a template for homology-directed repair of the double-stranded break produced by the CRISPR/Cas9 complex. The ultramer sequence (5′-GGTTAGATTGTGGGCGCCAGCATGAATTACTATGGGTTAGCCTAATGATCCAAAAATCTCTTTTAAGGTAGATCTGGTGC**A**GGAAACTTTGGTGGTGGTCGTGGAGGCGGTTTTGGTGGCAATGACAATTTTGGTCGAGGAGGGAACTTC-3′) corresponded to the genomic sequence evenly flanking amino acid S199 but contained a 6-base substitution that: 1) introduced the S199A mutation, 2) introduced a BglII restriction site to facilitate genotyping, and 3) mismatches in the seed sequences of the single-guide RNA (sgRNA) to prevent further editing of the mutant allele by Cas9/sgRNA complex. To generate the mice, pronuclear-stage C57BL/6J zygotes were injected with a mixture of Cas9 mRNA (100 ng/µl), Hnrnpa1-sgRNA1F (50 ng/µl), and Hnrnpa1-S199A Ultramer (Innovative Technologies Development Core) (0.5 µM) by the transgenic core at the University of Pittsburgh’s department of immunology. From the injected zygotes, 185 embryos developed to the two-cell stage and were transferred to the oviducts of six CD1 pseudo-pregnant female recipients; 18 live pups were born, several of which carried the targeted allele. Two founders were selected and backcrossed to WT C57BL/6 to generate independent lines. Subsequently, mice heterozygous for the A1 mutation were bred to generate A1-MUT and WT littermates. Both male and female mice were analyzed. For genotyping the target locus was amplified from genomic DNA with the following primers: Hnrnpa1-F1, 5′-TGACTGACAGAGGCAGTGGG-3′, and Hnrnpa1-R1, 5′-AATGCTGGAAACACAGGGTAGC-3′. The PCR products were subjected to RFLP analysis with BglII, WT product is 518 bp, and the S199A products are digested into fragments of 350 and 168 bp.

### Mice

B6.SJL-*Ptprc^a^ Pepc^b^*/BoyJ, (B6 CD45.1) and B6.Cg-Tg(TcraTcrb)425Cbn/J (OTII) mice were purchased from The Jackson Laboratory. OTII mice from The Jackson Laboratory were bred with A1-MUT to generate heterozygous mice for the A1 mutation and OTII. The OTII A1-HET mice were then bred together to generate OTII A1-MUT and OTII A1-WT littermates. All of the mice were housed in a specific pathogen-free facility at the University of Pittsburgh and were treated under Institutional Animal Care and Use Committee–approved guidelines in accordance with approved protocol.

### Verification of the impact of the S199A mutation by Western blot

CD4^+^ cells were isolated using a negative isolation kit (Miltenyi Biotec) from WT and A1-MUT mice. 2–2.5 × 105 CD4^+^ T cells were plated in a 96-well plate (Falcon REF 353077) with (1.0 µg/ml) plate-bound anti-CD3 in the presence of soluble (1.0 µg/ml) anti-CD28 for 30 min. The cells were lysed in ice-cold lysis buffer (Cell Signaling) with protease inhibitor (Thermo Fisher) on ice for 40 min. The supernatant from the cell lysate was precleared with protein G beads (Thermo Fisher) for 30 min. Lysates were incubated with anti-hnRNP A1 (D21H11; Cell Signaling) Ab at 4°C overnight. The hnRNP A1 complexes were captured by protein G–coated beads. As a negative control, cell lysate treated with IgG (G3A1; Cell Signaling) Ab was incubated with protein G beads. The beads were washed four times with cell lysis buffer. The captured protein was eluted with SDS and heated at 95°C for 5 min. Western blotting was performed using an Ab that recognizes P-Akt RXRXXS/T (23C8D2; Cell Signaling), and imaged. Then, the same blot was stripped and probed for hnRNP A1(D21H11; Cell Signaling) for quantification.

### Immunoprofiling, cell staining, and flow cytometry

Spleen, thymus, bone marrow, mesenteric lymph nodes, and lymph nodes (axillary, brachial, and inguinal were pooled) were isolated from mice aged 6–8 wk. RBCs from the spleen and bone marrow were lysed in ACK lysing buffer (Thermo Fisher) for 2 min and then washed in FACS buffer.

Single-cell suspensions were stained with Abs for: TCRβ-BV605 (H57-597; BD Biosciences), CD4-BUV395 (GK1.5; BD Biosciences), CD8a -PE-Cy5 (53-6.7; Thermo Fisher), CD62-BV650 (MEL-14; BD Biosciences) GITR-BUV661 (DTA.1; BD Biosciences), PD-1-PE-Dazzle594 (PMP1-30; BioLegend), CD25-Alexa Fluor 700 (PC61.5; BioLegend), CD44-BV510 (IM7; BD Biosciences), GARP-PE-Cy7 (F011-5; BioLegend), NRP-1-PE (3D5304M; BioLegend), Helios-APC eFlour780 (22F6; Thermo Fisher), Foxp3-Alexa Fluor 450 (FJK-19S; Thermo Fisher), T-BET-Percp-Cy5.5 (4B10; Thermo Fisher), GATA3-PE (L50-823; BD Biosciences), RORyT-APC (AFKJS-9; Thermo Fisher), GR1-Alexa Fluor 532 (RB6-8C5; Thermo Fisher), CD21/35-BV510 (7E9; BioLegend), CD127-FITC (SB/199; Thermo Fisher), MHCII-BV785(M5/114.15.2; BioLegend), CD45.1-BV421 (A20; BD Biosciences), CD45.2-BUV805 (104; BD Biosciences), B220-FITC (RA3-6B2; Thermo Fisher), CD11b -APC-ef780 (M1/70; Thermo Fisher), CD11c (N418; BioLegend), CD23-Pe-Dazzle594 (B3B4; BioLegend), F4/80-BV510 (BM8; BioLegend), pDCA1-BV650 (927; BioLegend), CD138-BV605 (281-2; BD Biosciences), NK1.1-PerCP-Cy5.5 (PK136; Thermo Fisher), CD27-APC (LG.7F9; Thermo Fisher), CD117-Pe-Cy7 (2BB; Thermo Fisher), CD19-BV480 (1D3; BD Biosciences), CD93-PE (AA4.1; Thermo Fisher), ICOS-FITC (DX29; BioLegend), CXCR5-PE (RF8B2; BD Biosciences), CD38- PerCP-Cy5.5 (HIT2; BD Bioscience), CD95- PE-Cy7(DX2; Thermo Fisher), CXCR4-BV421 (12G5; BD Biosciences), CD86-PE (24F;BioLegend), IgG1-FITC (RMG1-1; BioLegend), NP-APC, and BCL6-PeCy7 (K112-91; BD Bioscience). Dead cells were discriminated by staining with Zombie NIR dye (BD Bioscience) with surface staining on ice for 30 min in PBS. For intracellular staining of transcription factors, the cells were stained with surface markers, fixed in Fix/Perm buffer (eBioscience) overnight, washed twice in permeabilization buffer (eBioscience), and stained in permeabilization buffer for 2 h on ice. The samples were acquired on the Aurora (Cytex) and analyzed by FlowJo (Tree Star). For the identification of various immune cell populations, we first subgated on live single cells. From this gate, the gating strategy for each T cell population is shown in Supplemental Fig. 3.

### In vitro skewing assays

Naive CD4^+^ cells were isolated using a negative isolation kit (Miltenyi Biotec) from WT and A1-MUT mice. Anti-CD3 was coated on a plate for 2 h at 37°C. In high versus low skews, 2–2.5 × 10^5^ naive CD4^+^ T cells were plated in a 96-well plate (Falcon) under low-dose (0.25 µg/ml) or high-dose (1.0 µg/ml) plate-bound anti-CD3 (17A2; Thermo Fisher) in the presence of soluble (1.0 µg/ml) anti-CD28 (37.51; Thermo Fisher). The cells were harvested after 5 d. In cytokine skews of 2–2.5 × 10^5^, naive CD4^+^ T cells were stimulated under the following conditions: all cells (1.0 µg/ml of plate-bound anti-CD3 and 1.0 µg/ml of anti-CD28); Th0 (10 µg/ml of anti-IFNγ, 10 µg/ml of anti-IL-4, and 20 U/ml of IL-2); Th1 (10 ng/ml of IL-12, 10 µg/ml of anti-IL-4, and 20 U/ml of IL-2); Th2 (10 ng/ml of IL-4, 10 µg/ml of anti-IFNγ, and 20 U/ml of IL-2); Th17 (2.5 ng/ml of TGF-β, 20 ng/ml of IL-6, 10 µg/ml of anti-IFNγ, 10 µg/ml of anti-IL4, and 20 U/ml of anti-IL-2); and iTregs (5 ng/ml of TGF-β, 100 U/ml of IL-2, 10 µg/ml of anti-IFNγ, and 10 µg/ml of anti-IL4). The cells were harvested after 3 d.

### OVA tolerance model

Naive CD4^+^ cells were isolated (Miltenyi Biotec), and CFSE (Thermo Fisher) labeled from OTII × WT versus OII × A1-MUT mice on a CD45.2^+^ background. A total of 1 × 10^6^ cells were adoptively transferred to CD45.1^+^ mice. At 24 h post-transfer, the mice were fed OVA-containing mouse chow (10 mg/kcal) (TD.130362; ENVIGO) or standard chow for 7 d; then the mesenteric lymph node (MLN) and small intestine (SI) were isolated and assessed by flow cytometry.

### Cell isolation from the small intestine

The protocol for cell isolation from the SI was adapted from the work of Oldenhove et al. (30). Briefly, the intestines were removed from the mice, and Peyer’s patches and fat were removed from the SI. The intestine was cut (butterfly) open and rinsed with PBS on a petri dish to remove the mucus and stool. The SI was cut into 0.5–1-cm pieces and placed in DTT^+^ EDTA medium to rotate for 20 min at 37°C. The tissue was then rinsed in shake media, chopped into fine pieces, and digested in DNase + liberase medium (0.05% DNase, 100 µg/ml liberase TL; Roche) to rotate at 37°C for 25 min. Then complete, 3% FBS medium was added to stop the digestion. Single cells were sorted using a 70-µm mesh.

### NP^−^ immunization

Mice 8–12 wk of age were immunized i.p. with 100 µg of NP*^−^* keyhole limpet hemocyanin (NP-KLH) precipitated in Alum (31). On day 10 postimmunization, the spleens were isolated and assessed via flow cytometry.

### Quantification and statistical analysis

The data are presented as mean ± SEM, including n for each experiment, representing the number of mice used per group unless otherwise stated. Statistical significance was determined using unpaired Student *t* test when comparing two groups and one-way ANOVA with multiple comparisons when comparing various groups. All statistical analyses were calculated using Prism software (GraphPad).

## Results

### hnRNP A1-S199A does not affect lymphocytes at steady state

**FIGURE 1.**
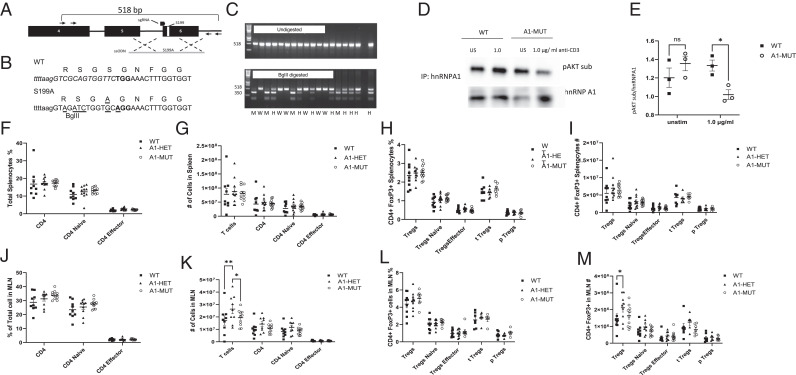
hnRNP A1-S199A does not affect lymphocytes at steady state. (**A**) Genomic structure of the region of interest of mouse Hnrnpa1. Exons are in black boxes, and the positions of S199 and of the sgRNA target sequence are depicted. Specific primers for PCR are represented by black arrows. Black dashed crosses represent the region of homology between the ssODN in gray and the genomic sequence. (**B**) Details of the substitutions in the ssODN that are introduced to create the S199A allele. The sgRNA target sequence is in italics in the WT sequence, the PAM sequence is in bold, substitution in the ssODN are underlined, and intronic sequences are in lowercases. (**C**) The genotyping results of the founder mice homozygous mutant (M) heterozygous (H) and homozygous wild type (W). (**D**) Immunoprecipitation (IP) for hnRNP A1 in CD4 T cells stimulated with anti-CD3 and anti-CD28 1.0 µg/ml for 30 min (1.0) versus unstimulated (US) cells followed by Western blot for p-AKT substrate. (**E**) The quantification of p-AKT substrate. (**F**–**M**) Immunocharacterization of the spleen (F–I) and MLNs (J–M) at steady state. Percentage (F and J) and absolute number (G and K) of CD4^+^ T cells, CD4^+^ naive (CD62L^+^ and CD44^−^) and CD4^+^ effector (CD62L^−^ and CD44^+^). Percentage (G and K) and absolute number (I and M) of Tregs (CD25^+^ Foxp3^+^), naive Tregs (CD62L^+^ and CD44^−^), effector Tregs (CD62L^−^ and CD44^+^), thymus-derived Tregs (t Tregs) (Helios^+^ and NRP-1^+^), and periphery-derived Tregs (p Tregs) (Helios^−^, NRP-1^−^). The data in (D and E) are representative of three experiments (*n* = 1 mice per group). The data in (F–M) represent the combination of eight experiments (*n* = 1–2 mice per group). Unpaired two-tail Student *t* test (E) and two-way ANOVA analysis with Bonferroni or Tukey post-test (F–M) were performed. *p < 0.05, **p < 0.01, ***p < 0.001.

Our previous work demonstrated that hnRNP A1 was phosphorylated by Akt under low TCR Ag stimulation that induced Treg differentiation. To explore whether Akt phosphorylation of hnRNP A1 was important for Treg induction and function, a new mouse strain (A1-MUT) was generated using CRISPR-Cas9 with a point mutation in the previously described Akt phosphorylation S199 site (27). To introduce the mutation in the locus, a 150-bp ssODN containing a 6-bp substitution that introduced the S199A mutation and created a BgIII restriction site for genotyping was used. The genomic diagram of the mutated region of mouse hnRNP A1 is shown (Fig. 1A), along with the DNA sequence of the substitution in the ssODN used to create the S199A allele (Fig. 1B). PCR was used to confirm the generation of the founder mice, and representative genotyping results are shown (Fig. 1C). The founder mice were bred as heterozygotes to produce A1-MUT, which are S199A/S199A mice along with WT littermate controls, which are S199/S199.

To determine whether the introduction of the Ser to Ala point mutation had an impact on the ability of Akt to phosphorylate hnRNP A1, T cell activation assays were performed with WT and A1-MUT mice. CD4^+^ T cells were isolated and stimulated in vitro with anti-CD3 (1.0 µg/ml) and anti-CD28 (1.0 µg/ml) for 30 min. Cell lysates were prepared, and immunoprecipitates of hnRNP A1 were generated. Western blot analysis of the immunoprecipitates using an Ab that recognizes phosphorylated Akt substrates revealed a significant reduction in the phosphorylation of mutated hnRNP A1 by Akt following activation (Fig. 1D, 1E).

To determine the impact of the hnRNP A1-S199A mutation on immune populations at steady state, a detailed characterization of the percentage and number of immune cells in primary and secondary lymphoid organs using spectral flow cytometry was performed. There were subtle increases in the percentage of marginal zone B cells in the spleen of the A1-HET mice, as well as an increase in the present and number of dendritic cells in the lymph nodes. There were no significant changes in the phenotype or numbers of other B cells subsets, dendritic cell subsets, macrophages, neutrophils, or NK cells at steady state (Supplemental Fig. 1). T cell development in the thymus was examined by assessing the CD4 SP, CD8 SP, DN, and DP populations (Supplemental Fig. 2), and no significant changes in the number or phenotype of any of the standard thymic populations were observed during T cell development. In the spleen, there were no significant changes in the CD8^+^ T cell (Supplemental Fig. 3) or overall CD4^+^, CD4^+^ naive (CD62L^hi^ CD44^lo^), CD4^+^ effector (CD62L^lo^ CD44^hi^) and Tregs (CD25^hi^ Foxp3^+^) percentages (Fig. 1F), or number (Fig. 1G). There was an increase in the number of CD8^+^ T cells in the MLN of the A1-HET.

To further assess Treg development at steady state, a more in-depth analysis of the Tregs was performed by assessing Treg (TCRβ^+^, Foxp3^+^), subpopulations including naive Tregs (CD62L^hi^ CD44^lo^), effector Tregs (CD62L^lo^ CD44^hi^), thymus-derived Tregs (tTregs) (Helios^+^, NRP-1^+^), and periphery-derived (pTregs) (Helios^−^, NRP-1^−^). There were no significant differences in the percentages (Fig. 1H) or number (Fig. 1I) of Treg subsets in the spleen. In the MLNs, there were no significant changes in CD8^+^ T cells (supplemental Fig. 3) or overall CD4^+^, CD4^+^ naive, CD4^+^ effector, and Treg percentages (Fig. 1J), but a significant increase in the total number of T cells in the hnRNP A1-S199A heterozygotes (A1-HET) (Fig. 1K) was observed. Within the Tregs, there were no significant changes in the percentage of Treg subsets (Fig. 1L). A significant increase in total Treg numbers was observed in the A1-HET (Fig. 1M). These results suggest that the hnRNP A1-S199A mutation does not have a major impact on immune cells at steady state, but the changes in the MLN warranted further investigation.

### hnRNP A1-S199A does not affect T cell differentiation in vitro

**FIGURE 2.**
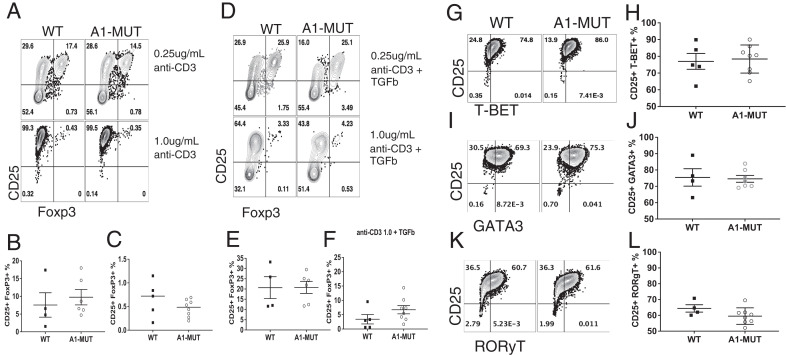
hnRNP A1-S199A does not affect CD4^+^ T cell differentiation in vitro. (**A**–**F**) Naive T from WT and A1-MUT cells were stimulated with a 1.0 µg/ml of anti-CD28 and a high dose 1.0 µg/ml or a low dose 0.25 µg/ml of anti-CD3. (A) Representative flow plot of induced Tregs. (B and C) Percentage of Foxp3^+^ Tregs under low-dose stimulation (B) and high-dose stimulation (C). Naive T cells stimulated with 10 ng/ml of TGF-β, 1.0 µg/ml of anti-CD28 and a high dose (1.0 µg/ml) or a low dose (0.25 µg/ml) of anti-CD3. (D) Representative flow plot of induced Tregs. (E and F) Percentage of Foxp3^+^ Tregs under low-dose stimulation (E) and high-dose stimulation (F). Naive T from WT and A1-MUT cells were stimulated with 10 ng/ml of IL-12, 10 µg/ml of anti–IL-4, and 20 U/ml of IL-2 to induce Th1. (**G** and **H**) Representative flow plot of T-Bet^+^ CD4^+^ T cell, stimulated under 1.0 µg/ml anti-CD3 and CD28 (G) and the percentage (H). Naive T from WT and A1-MUT cells were stimulated with 10 ng/ml of IL-4, 10 µg/ml of anti-IFNγ, and 20 U/ml of IL-2 to induce Th2. (**I** and **J**) Representative flow plot of GATA3 ^+^ CD4^+^ skewed T cells (I) and the percentage (J). Naive T from WT and A1-MUT cells were stimulated with 2.5 ng/ml of TGF-β, 20 ng/ml of IL-6, 10 µg/ml of anti-IFNγ, 10 µg/ml of anti-IL4, and 20 U/ml of anti-IL-2 to induce Th17. (**K** and **L**) Representative flow plot (K) of RORγT^+^ CD4^+^ skewed T cells and the percentage (L). The data in (B, C, E, F, H, J, and L) represent the combination of four independent experiments (*n* = 1–2 mice per group). Unpaired two-tail Student *t* test was performed. *p < 0.05, **p < 0.01, ***p < 0.001.

We can replicate the signal strength of a low-affinity Ag by using low-dose anti-CD3 (17). Previous work has shown that Akt phosphorylates hnRNP A1 under low TCR stimulation, and knockdown of hnRNP A1 via siRNA resulted in reduced Treg differentiation in vitro (18). CD4^+^ T cells from WT and A1-MUT spleens were cultured in vitro to induce Th1, Th2, Th17, or Tregs. Previous studies have shown low doses (0.25 µg/ml anti-CD3 and 1.0 µg/ml anti-CD28) skew toward Tregs, whereas high doses (1.0 µg/ml anti-CD3 and 1.0 µg/ml anti-CD28) skew toward Th1 in B6 mice. To investigate the role Akt phosphorylation of hnRNP A1 has on naive CD4 T cell differentiation, a high- versus low-dose stimulation was performed by isolating and stimulating 2 × 10^5^ naive CD4^+^ T cells/well in a 96-well plate from WT versus A1-MUT with a low versus high stimulation (Fig. 2A–C). No changes in Treg induction under low-dose stimulation (Fig. 2B) or high-dose stimulation harvested at day 5 (Fig. 2C) were observed. To increase the number of Tregs induced, TGF-β, a known cytokine important in Treg induction (32–34) was added to the culture (Fig. 2D–F); no changes in Treg induction under low-dose stimulation (Fig. 2 E) or high-dose stimulation (Fig. 2F) in the presence of TGF-β (5 ng/ml) were observed. Under high-dose stimulation, there were no differences in Th1 cell induction (Fig. 2G, 2H). No significant differences in the number of Th2 (Fig. 2I, 2J) and Th17 cells (Fig. 2K, 2L) were observed following culture of A1-MUT and WT CD4^+^ T cells in the presence of skewing cytokines.

### hnRNP A1-S199A does not affect tolerance in the gut

**FIGURE 3.**
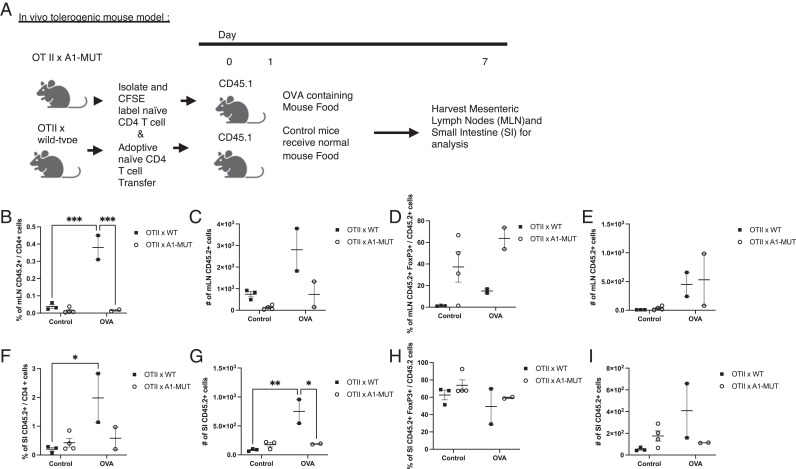
hnRNP A1-S199A does not affect tolerance in the gut. Using the in vivo tolerogenic model, naive T cells were isolated from OTII (WT) or OTII × hnRNP A1- S199A (OTII × A1-MUT) mice and CFSE labeled, and 1 × 10^6^ naive CD4 T cells were injected into CD45.1 mice. The mice were fed OVA mouse food for 7 d. Lymphocytes were isolated from the MLN and small intestine. (**A**) Diagram of OVA tolerogenic model. The images were generated using BioRender.com. (**B** and **C**) Percentage (B) and absolute number (C) of CD45.2^+^ cell in the MLN. (**D** and **E**) Percentage (D) and absolute number (E) of CD45.2^+^, Foxp3^+^ in the MLN. (**F** and **G**) Percentage (F) and absolute number (G) of CD45.2^+^ cell in the SI. (**H** and **I**) The percentage (H) and absolute number (I) of CD45.2^+^, Foxp3^+^ in the SI. The data in (B–I) are representative of five independent experiments (*n* = 2–4 mice per group). A two-way ANOVA analysis with Tukey post-test was performed. *p < 0.05, **p < 0.01, ***p < 0.001.

The initial characterization of the immune system at steady state revealed subtle changes in T and Treg cell numbers in the MLN. Many groups have shown the importance of Tregs in the SI for the maintenance of oral tolerance (5, 7). To investigate whether the A1-MUT had an impact on T cells in the gut, an in vivo tolerance model was used (35). In this model, naïve CD4^+^ T cells were isolated from CD45.2^+^ OTII (OTII^+^ × WT) or CD45.2^+^ OTII × hnRNP A1- S199A (OTII^+^ × A1-MUT) mice and CFSE labeled, and 1 × 10^6^ naive CD4 T cells were injected into CD45.1^+^ mice. Experimental mice were fed OVA mouse food for 7 d, whereas control mice were fed normal mouse chow. After 7 d, lymphocytes were isolated from the MLN and SI (Fig. 3A). In the MLN, there was an increase in the percentage of OVA-fed OTII^+^ × WT CD45.2^+^ cells when compared with both control and OTII^+^ × A1-MUT CD45.2^+^ cells (Fig. 3B), but no significant difference in the number (Fig. 3C) of CD45.2^+^ cells was observed in mice fed OVA chow. There were no differences in the percentages (Fig. 3D) or numbers (Fig. 3E) of CD45.2^+^ Tregs in the MLN. In the SI, there was an increase in the percentage of OTII^+^ × WT CD45.2^+^ cells mice fed OVA chow when compared with control, which was not seen with OTII^+^ × A1-MUT CD45.2^+^ cells (Fig. 3F). There was a significant increase in the number of OTII^+^ × WT CD45.2^+^ cells in mice fed OVA chow when compared with control and OTII^+^ × A1-MUT CD45.2^+^ (Fig. 3G). There were no differences in the percentages (Fig. 3H) or numbers (Fig. 3I) of CD45.2^+^ Tregs in the MLN.

### hnRNP A1-S199A does not affect T follicular helper cell induction or germinal center formation

**FIGURE 4.**
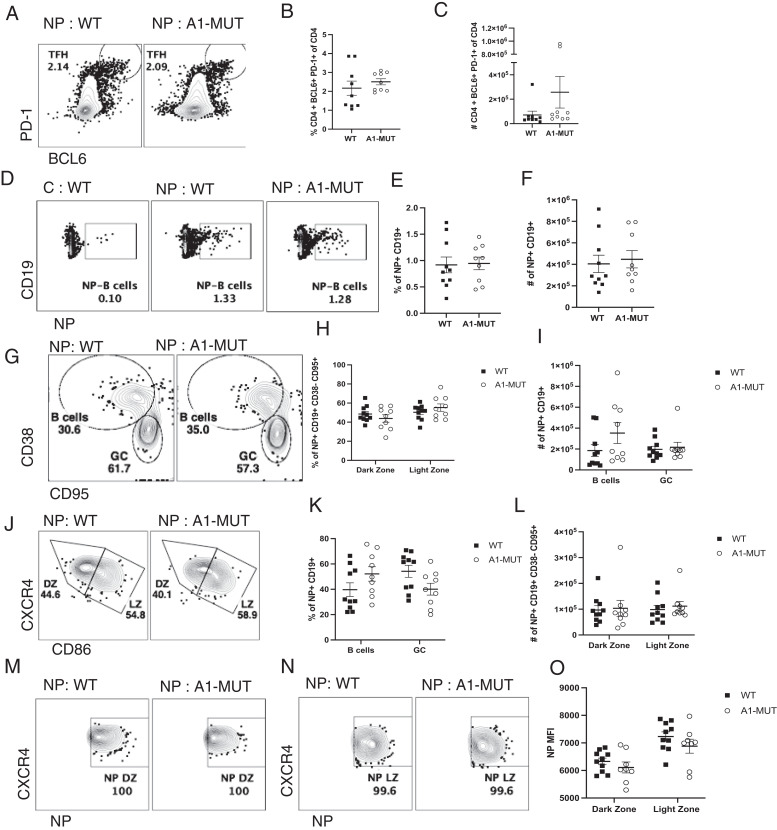
hnRNP A1-S199A does not affect T follicular helper cells induction or germinal center formation. Using the NP immunization model. WT or A1-MUT mice were immunized with 100 μg of NP-KLH or nonimmunized (control [C]), and then the spleen was harvested on day 10 postimmunization. (**A**) Representative flow plot of T follicular helper (TFH) cells (PD-1^+^ BCL6^+^). (**B** and **C**)Percentage (B) and absolute number (C) of TFH. (**D**) Representative flow plot of total NP^+^ B cells in unimmunized WT (*left*), immunized WT (*middle*), and immunized A1-MUT (*right*) mice. (**E** and **F**) Percentage (E) and absolute number (F) of NP^+^ B cells in the spleen of immunized WT and A1-MUT mice. (**G**) Representative flow plot of B cells (CD38^+^ CD95^−^) versus GC B cells (CD38^−^ CD95^+^) within the NP-specific population. (**H** and **I**) Percentage (H) and absolute number (I) of NP^+^ GC B cell in the dark and light zone of the GC. (**J**) Representative flow plot of dark and light zone in the GC. (**K** and **L**) Percentage (K) of dark (CD184^hi^ CD86^lo^) and light zone (CD184^lo^ CD86^hi^) GC B cells and absolute number (L). (**M** and **N**) Representative flow p lot of NP^+^ B cell in the dark zone (M) and light zone (N). (**O**) NP MFI of NP^+^ dark and light zone cells. The data in (B, C, E, F, H, I, K, and L) represent the combination of three independent experiments. The results represent the means ± SEM of three independent experiments. Unpaired two tail Student *t* test and two-way ANOVA analysis with Tukey post-test were performed. *p < 0.05, **p < 0.01, ***p < 0.01.

To determine whether the hnRNP A1 S199A mutation influences the ability of Tfh cells to drive GC formation, the NP-KLH immunization model was used (31). Because the hnRNP A1 S199A mutation is present in every cell, not just CD4^+^ T cells, the NP-KLH immunization model allowed us to examine the function of both Tfh and B cells. WT and A1-MUT mice were immunized with 100 µg of NP-KLH, and the spleen was harvested on day 10 postimmunization. There were no significant differences in the percentage (Fig. 4A, 4B) or number (Fig. 4C) of Tfh (PD-1^+^, BCL6^+^) cells. No significant differences in the percentage (Fig. 4D, 4E) and number (Fig. 4F) of NP^+^ B cells (NP^+^, CD19^+^) were observed. Within the NP^+^ B cells, there were no significant differences in the percentage (Fig. 4G, 4H), and the number (Fig. 4I) of NP^+^ B cells (CD38^+^, CD95^−^) versus GC B cells. The GC B cells were further divided in the light zone (CD184^lo^, CD86^hi^) versus the dark zone (DZ) (CD184^hi^, CD86^lo^) based on CD184 (CXCR4) and CD86 expression (Fig. 4J). No significant differences in the percentage (Fig. 4J, 4K) and number (Fig. 4L) of NP^+^ dark zone and light zone GC B cells were observed. We noticed a slight change in the NP MFI in the dark (Fig. 4M) and light zone (Fig. 4N), but ultimately this was not statistically significant (Fig. 4O).

## Discussion

The experiments presented here describe a new mouse model in which the Akt phosphorylation site at S199 in hnRNP A1 was mutated to alanine. Using the hnRNP A1-S199A mice, we determined that reducing phosphorylation of hnRNP A1-S199 did not affect the immune system at steady state. We determined that CD4 T cell differentiation, specifically Treg induction was not primarily dependent on Akt phosphorylation of hnRNP A1-S199, nor did it affect the generation of Tfh and the formation of NP-specific GC in an immunization model. We did observe a reduction in the ability of A1-MUT T cells to migrate and stay in the MLN suggesting a potential homing defect, but more research is needed to establish the mechanism. Finally, although we did not identify a role for phosphorylation of hnRNP A1 at S199 in T cell differentiation and function, this mouse model can be used to study phosphorylation of hnRNP A1 in other cell types and disease situations.

Many groups have shown that post-translational modification of hnRNP A1, such as phosphorylation can alter hnRNP A1’s function (28, 36–40). Our previous studies identified hnRNP A1 as a potential target for Akt phosphorylation under low-dose stimulation, and using siRNA to knockdown hnRNP A1, we showed a reduction in iTreg induction (18). A previous study showed, using an in vitro phosphorylation assay with purified Akt and hnRNP A1, that S199 was the unique site on hnRNP A1 phosphorylated by Akt (27, 28). Based on this study, we generated the S199A hnRNP A1 mutant to further examine the effects of Akt phosphorylation of hnRNP A1 on iTreg development. CD4 T cells expressing the A1-MUT showed a reduction in Akt phosphorylation of hnRNP A1 following TCR activation, confirming that this site is phosphorylated as a result of TCR engagement. However, the reduction was only partial, and baseline phosphorylation of hnRNP A1 was observed in WT and AI-MUT T cells. The lack of complete loss of hnRNP A1 phosphorylation could be the result of differences between the models. As stated previously, the S199 phosphorylation site was identified using purified activated Akt and hnRNP A1 in vitro and verified in serum-stimulated 293 cells overexpressing either wild-type hnRNP A1 or A1-S199A GST fusion protein (27), whereas our in vivo studies likely involve more complex interactions including additional phosphorylation sites. Other studies have shown different sites on hnRNP A1 (S4 and S6) (41) that are potentially phosphorylated by Akt. It is possible that hnRNP A1 S4 or S6 is Akt’s primary phosphorylation site in T cells that drives Treg induction. It is also possible that a combination of phosphorylation site or all three sites, S4, S6 (41), and S199, are needed for T cells to differentiate into Treg; thus, additional sites may need to be mutated to prevent Akt phosphorylation of hnRNP A1 completely.

The importance of hnRNP A1 in human Treg differentiation was recently demonstrated in a study in which knockdown of hnRNP A1 via shRNA in human T cells led to reduced Foxp3 expression and suppressor function (37) Further, this study showed, by overexpressing hnRNP A1 S199A versus hnRNP A1 S199D in HEK 293T cells, that phosphorylation of hnRNP A1 S199 was essential for the formation of Foxp3- hnRNP A1 complexes (37) This study confirms our previous observation that knockdown of hnRNP A1 reduces Treg differentiation and function and provides a novel mechanism by which phosphorylation of hnRPNA1 at S199 affects Foxp3 expression and function. These association studies were performed using overexpression of constructs in a cell line, whereas our model relies on Akt naturally phosphorylating endogenous levels of hnRNP A1 in T cells under stimulation.

At steady state, there were no significant changes in the CD4^+^ T cell or CD8^+^ T cell subsets in the spleen, but we observed an increase in the total number of T cells and Treg in the MLN of A1-HET mice. The changes observed at steady state in the MLN prompted us to further investigate T cell subsets in the MLN and gut. Many groups have shown Tregs can be induced in the gut using an OVA oral tolerance model (35, 42), and we used this model to assess the ability of A1-MUT OTII^+^ T cells to differentiate into Treg in vivo. There was no significant difference in Treg induction in the MLN or the SI using the OVA oral tolerance model between WT and A1-MUT OTII T cells. However, in both the MLN and the SI, we do observe an increase in the percentage of OTII^+^ × WT CD45.2^+^ CD4^+^ T cells in response to OVA. No increases in OTII^+^ CD4^+^ T cells were observed in the OTII^+^ × A1-MUT, suggesting that the A1- MUT T cells are unable to respond to OVA at the same levels as WT. This also suggests a potential defect in OTII-A1 MUT T cell’s ability to migrate to the MLN. One group suggests that the ubiquitylation of hnRNP A1 plays a role in cell migration in EGF signaling (43). The migration defect in the OTII-A1 MUT T cells may warrant further investigation.

The hnRNP A1 S199A mutation is in every cell, not just in CD4^+^ T cells, and the NP-KLH immunization model allowed us to determine the effect of the hnRNP A1 S199A mutation on Tfh differentiation, GC formation, and the generation of high affinity NP-specific B cells. The optimal production of high-affinity Ab is dependent on the formation of the GC. In the GC, there are two zones: the light zone and the dark zone. In the dark zone, B cells proliferate and undergo somatic hypermutation, whereas in the light zone, Tfh positively select high-affinity GC B cells (12, 44–46). NP-KLH is a T cell-dependent Ag effective in generating GC (46). Previous studies have shown that this model can be used to disrupt the Tfh induction under certain conditions (47). Using the NP-KLH model, no changes in A1-MUT Tfh numbers or GC formation were observed. While assessing the GC B cells in this study, a slight reduction in NP MFI was observed in A1-MUT GC B cells; although not statistically significant, it does suggest that affinity maturation in mutant B cells may not be optimal. Another RBP hnRNP F has been shown to regulate alternative splicing of CD40, important for GC B cell formation. This study also showed hnRNP A1 and hnRNP F antagonistically regulate alternative splicing of CD40 (48). These results suggest that hnRNP A1 plays a role in B cell function in the GC, and the role of hnRNP A1 S199A mutation in B cells merits further study.

Although this mutant mouse model did not reveal any strong phenotypes, there are other potential applications for the A1-MUT mouse model, including the study of other kinases that are known to interact with hnRNP A1. PKCζ was shown to phosphorylate hnRNP A1 (40). Another group examined PKA’s ability to phosphorylate hnRNP A1 at sites S4, S6, S36, S182, and S199 and determined S199 to be the sole phosphorylation site, which, when phosphorylated by PKA, suppresses annealing activity in HeLa cells (36). The A1-MUT mouse model could be used as a start for other site-directed mutagenesis of hnRNP A1 examining the role of phosphorylation by other kinases in other systems or cell types.

RBP are complex proteins that often work in complexes, and there could be redundancy that explains the lack of a robust phenotype in this model. hnRNP A1 has transcriptional function by associating with several promoter sequences, including NF-κB (29, 49). hnRNP A1 can also associate with the spliceosome of several genes and mediate mRNA nuclear export (29, 50). Mutation in the hnRNP A1 S199 site results in a reduction in annealing activity in a glioblastoma cell line, and LN229 (28) could be further studied using this mouse model. In addition, mutation of the S199 site in hnRNP A1 was shown to disrupt association of hnRNP A1 with Foxp3 using overexpression in 293T cells (37). Although the focus of this paper is to understand the role Akt phosphorylation of hnRNP A1-S199 has on T cell differentiation and function, this mutation is on every cell, so this mouse model can be used to investigate phosphorylation of hnRNP A1-S199 in other cell types by other kinases. The hnRNP A1 S199A mutation is subtle but provides a basis for additional mutations as more phosphorylation sites are discovered.

## Supplementary Material

Supplemental Figures 1 (PDF)
