# Supplementary material for: Phosphorylation of hnRNP A1–Serine 199 Is Not Required for T Cell Differentiation and Function
Source: Immunohorizons. 2024 Feb 9;8(2):136–46. doi: 10.4049/immunohorizons.2300074 (PMC10916359; doi:10.4049/immunohorizons.2300074)
Supplement: Supplemental Figures 1 (PDF) [file IH_2300074_Supplemental_1.pdf]

Supplementary Figure 1

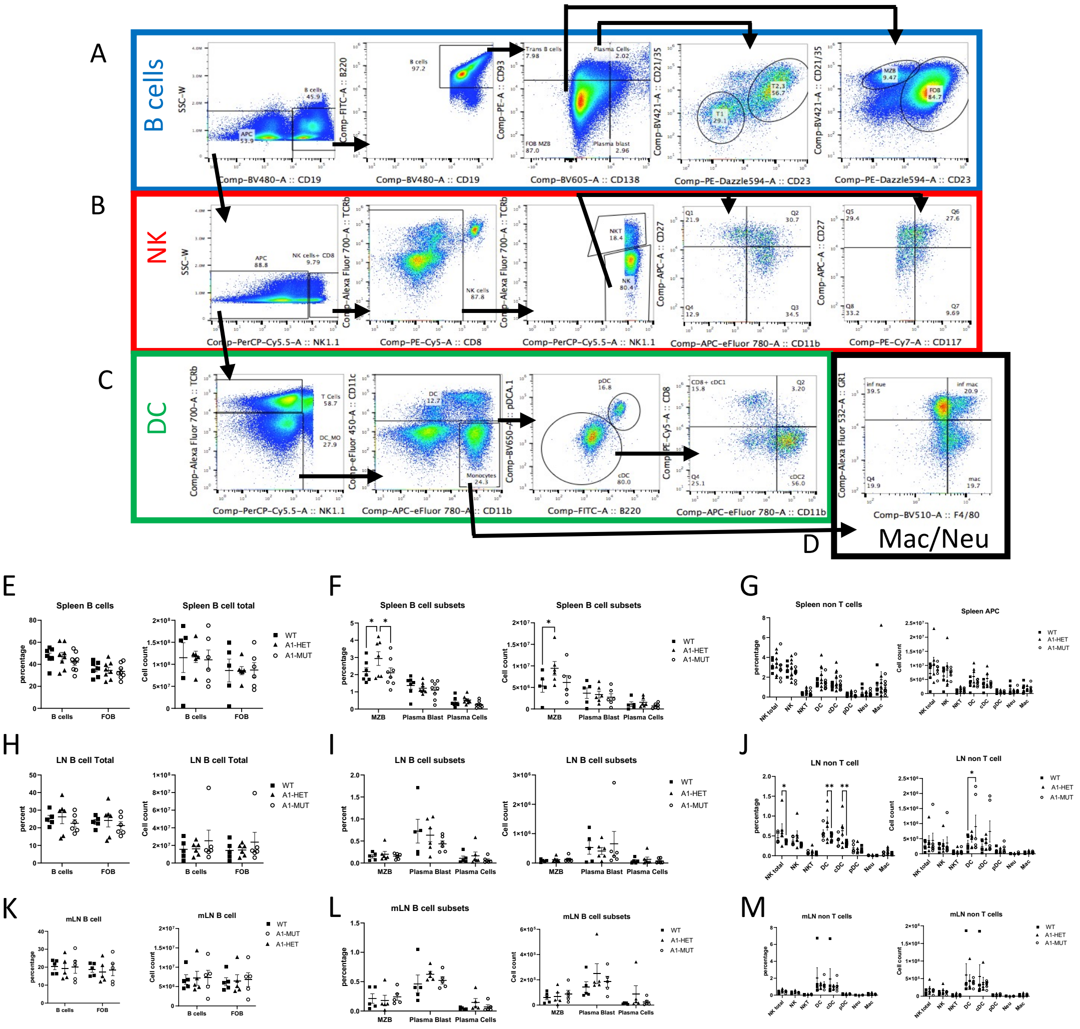


**Supplemental Figure 1: hnRNP A1-S199A does not affect lymphocytes at steady state.** Gating strategy and immuno-characterization of non-T cells at steady state. **(A-D)** Representative flow plot of gating strategy for non-T cell **(A)** The gating strategy used for B cells (blue), **(B)** natural killer cells (NK) (red), **(C)** Dendritic cells (DC) (green)**(D)** Macrophages (Mac) and Neutrophils (Neu) (black). **(E)** Percentage and absolute number of Total B cells (CD19$^+$ B220$^+$), follicular B cells (CD23$^+$ CD21/35$^+$) **(F)** marginal zone B cells (MZB), plasma blast, plasma cells **(G)** total NK, NKT, DC, cDC, pDC, Neu and Mac in the spleen.**(H)** Percentage and absolute number of Total B cells, follicular B cells **(I)** marginal zone B cells (MZB), plasma blast, plasma cells **(J)** total NK, NKT, DC, cDC, pDC, Neu and Mac in the lymph nodes. **(K)** Percentage and absolute number of total B cells, follicular B cells **(L)** marginal zone B cells (MZB), plasma blast, plasma cells **(M)** total NK, NKT, DC, cDC, pDC, Neu and Mac in the mesenteric lymph nodes. Data is representative of 8 experiments (n = 1-2 mice per group). Two-way ANOVA analysis with Bonferroni or Tukey post-test (F-M) was performed *p < 0.05, **p < 0.01, ***p < 0.001.

Supplementary figure 2

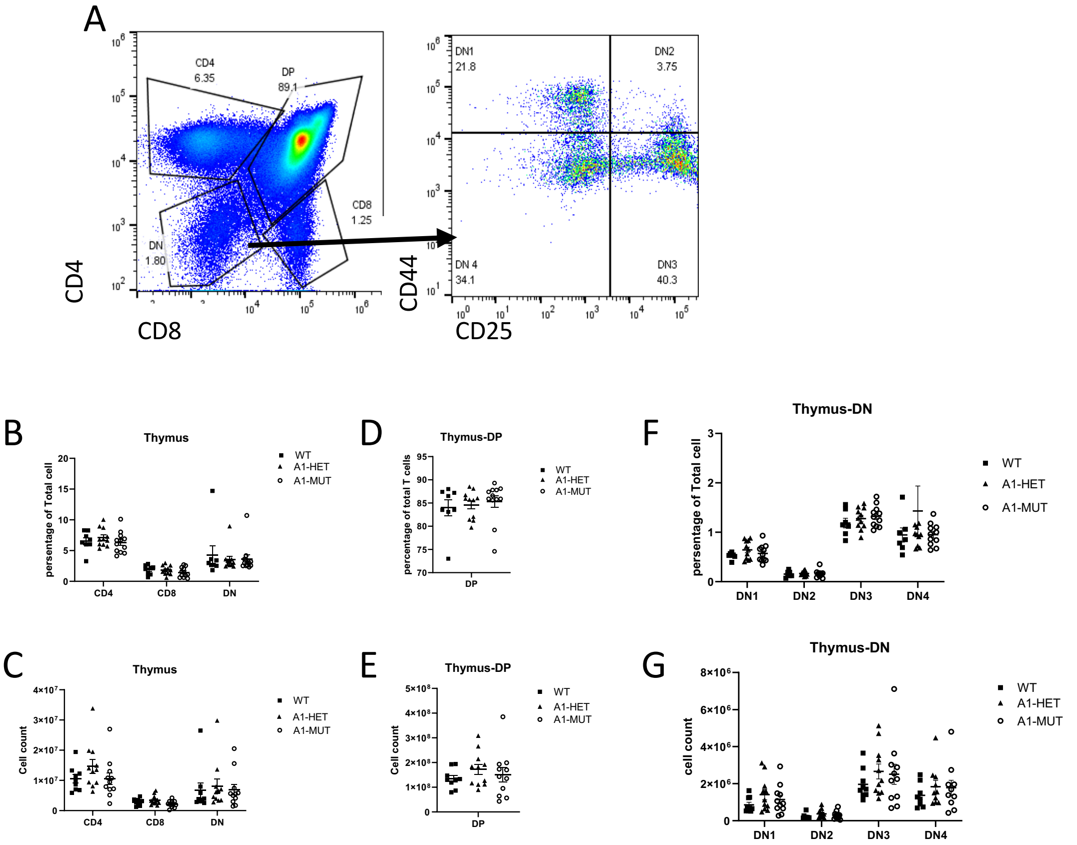


**Supplemental Figure 2: hnRNP A1-S199A does not affect T cell development at steady state.** Gating strategy and immuno-characterization of the thymus at steady state. Representative flow plot of thymus gating strategy. **(A)** The gating strategy for the thymus. **(B)** Percentage and **(C)** absolute number of $CD4^+$, $CD8^+$ and, DN cells. **(D)** Percentage and **(E)** absolute number of double positive (DP) thymocytes. **(F)** Percentage and **(G)** absolute number of DN subsets thymocytes. Data is representative of 7 experiments (n = 1-2 mice per group). Two-way ANOVA analysis with Bonferroni or Tukey post-test (F-M) was performed $*p < 0.05$, $**p < 0.01$, $***p < 0.001$.

Supplementary Figure 3

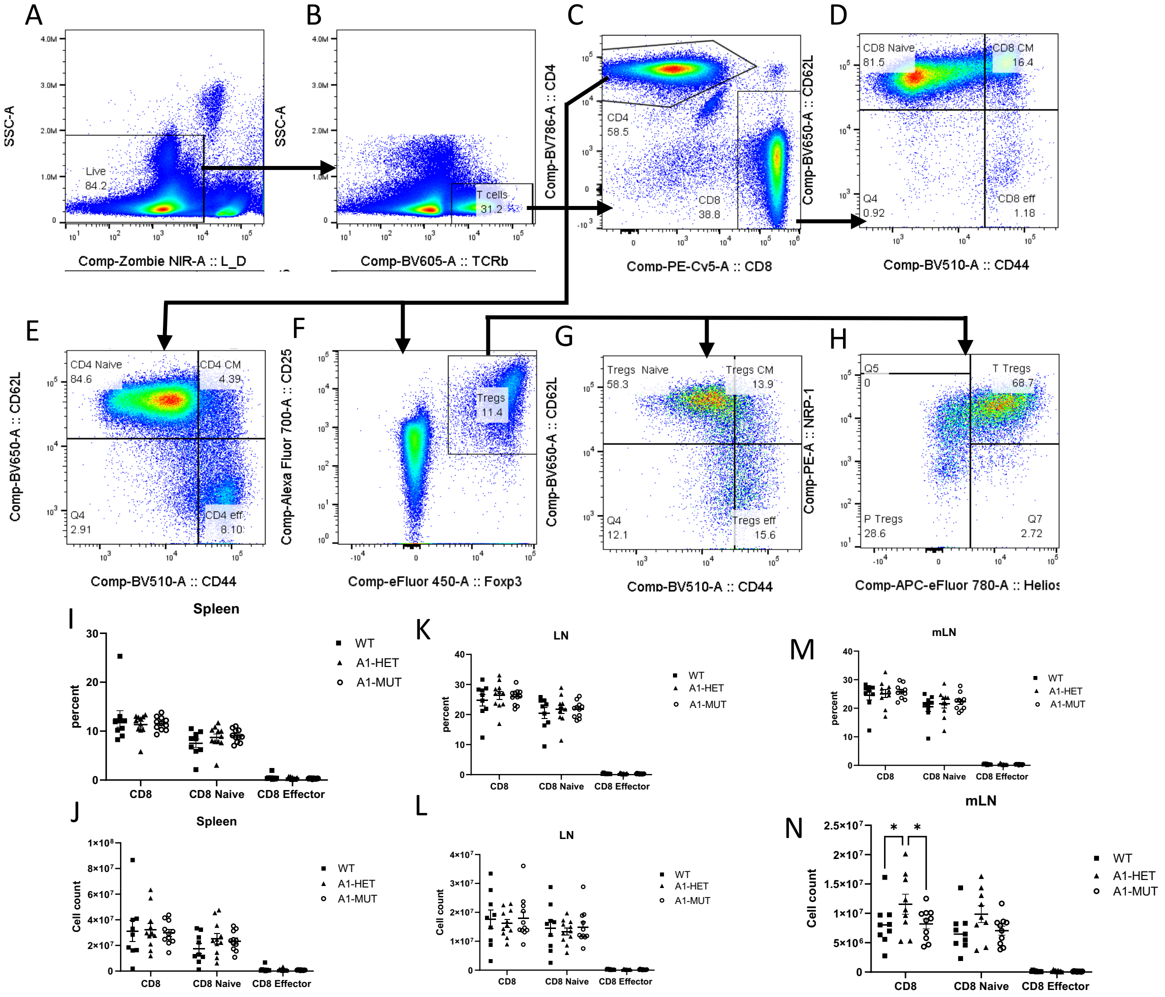


**Supplemental Figure 3: hnRNP A1-S199A does not affect CD8+ T cells at steady state.** Gating strategy and immuno-characterization of T cells at steady state. Representative flow plot of gating strategy for T cell. **(A)** Pre-gated on single splenocytes. **(B)** Pre-gated on live cells. **(C)** Pre-gated on T cells (TCRβ$^{+}$). **(D)** Pre-gated on CD8$^{+}$ T cells. **(E-F)** Pre-gated on CD4$^{+}$ T cells. **(G-H)** Pre-gated on Tregs (CD25$^{hi}$ Foxp3$^{+}$). **(I)** Percentage and **(J)** absolute number of CD8$^{+}$, naïve CD8$^{+}$ and effector CD8$^{+}$ T cells in the spleen. **(K)** Percentage and **(L)** absolute number of CD8$^{+}$, naïve CD8$^{+}$ and effector CD8$^{+}$ T cells in the lymph nodes (LN). **(M)** Percentage and **(N)** absolute number of CD8$^{+}$, naïve CD8$^{+}$ and effector CD8$^{+}$ T cells in the mesenteric lymph nodes (mLN). Data is representative of 8 experiments (n = 1-2 mice per group). Two-way ANOVA analysis with Bonferroni or Tukey post-test (F-M) was performed *p < 0.05, **p < 0.01, ***p < 0.001.
